# MicroRNA390-Directed *TAS3* Cleavage Leads to the Production of tasiRNA-*ARF3/4* During Somatic Embryogenesis in *Dimocarpus longan* Lour

**DOI:** 10.3389/fpls.2015.01119

**Published:** 2015-12-16

**Authors:** Yuling Lin, Lixia Lin, Ruilian Lai, Weihua Liu, Yukun Chen, Zihao Zhang, Xu XuHan, Zhongxiong Lai

**Affiliations:** Institute of Horticultural Biotechnology, Fujian Agriculture and Forestry UniversityFuzhou, China

**Keywords:** *Dimocarpus longan*, somatic embryo, microRNA390, *TAS3*-tasiRNA, auxin response factors, gene regulation

## Abstract

*Trans*-acting short-interfering RNAs (tasiRNAs) originate from *TAS3* families through microRNA (miRNA) 390-guided cleavage of primary transcripts and target auxin response factors (ARF3/-4), which are involved in the normal development of lateral roots and flowers in plants. However, their roles in embryo development are still unclear. Here, the pathway miR390-*TAS3*-*ARF3*/*-4* was identified systematically for the first time during somatic embryo development in *Dimocarpus longan*. We identified the miR390 primary transcript and promoter. The promoter contained *cis*-acting elements responsive to stimuli such as light, salicylic acid, anaerobic induction, fungal elicitor, circadian control, and heat stress. The longan *TAS3* transcript, containing two miR390-binding sites, was isolated; the miR390- guided cleavage site located near the 3′ end of the *TAS3* transcript was verified. Eight *TAS3*-tasiRNAs with the 21-nucleotides phase were found among longan small RNA data, further confirming that miR390-directed *TAS3* cleavage leads to the production of tasiRNA in longan. Among them, TAS3_5′D5+ and 5′D6+ tasiRNAs were highly abundant, and verified to target *ARF3* and *-4*, implying that miR390-guided *TAS3* cleavage with 21-nucleotides phase leading to the production of tasiRNA-ARF is conserved in plants. Pri-miR390 was highly expressed in friable-embryogenic callus (EC), and less expressed in incomplete compact pro-embryogenic cultures, while miR390 showed its lowest expression in EC and highest expression in torpedo-shaped embryos (TEs). *DlTAS3* and *DlARF4* both exhibited their lowest expressions in EC, and reached their peaks in the globular embryos stage, which were mainly inversely proportional to the expression of miR390, especially at the globular embryos to cotyledonary embryos (CEs) stages. While *DlARF3* showed little variation from the EC to TEs stages, and exhibited its lowest expression in the CEs stage. There was a general lack of correlation between the expressions of *DlARF3* and miR390. In addition, pri-miR390, *DlTAS3*, *DlARF3* and *-4* were up-regulated by 2,4-D in a concentration-dependent manner. They were also preferentially expressed in roots, pulp, and seeds of ‘Sijimi’ longan, implying their extended roles in the development of longan roots and fruit. This study provided insights into a possible role of miR390-tasiRNAs-ARF in plant somatic embryo development.

## Introduction

MicroRNAs and tasiRNAs are distinct classes of small RNAs, which control many aspects of development in plants by guiding silencing of target RNAs via cleavage or repression mechanisms (Chapman and Carrington, 2007). MiRNAs arise from endogenous transcripts that can form local fold-back structures, whereas tasiRNAs are generated by primary TAS transcripts that are initially targeted and sliced by miR173 (*TAS1* and *TAS2*), miR828 (*TAS4*) or miR390 (*TAS3*) (Pullman et al., 2003; Wilson et al., 2005; Axtell et al., 2006; Kikuchi et al., 2006; Montgomery et al., 2008b). The *TAS3* family is conserved in plants and has two sites that are complementary to miR390 (Krasnikova et al., 2013; Xia et al., 2013), while the *TAS1*, *TAS2*, and *TAS4* families are found only in *Arabidopsis* *thaliana* or close relatives, and each of them contain only a single site complementary to miR173 or miR828 (Pullman et al., 2003; Wilson et al., 2005; Axtell et al., 2006; Kikuchi et al., 2006; Montgomery et al., 2008b).

*TAS1* and *TAS2* tasiRNAs target PPR proteins (Kikuchi et al., 2006; Ruduś et al., 2006). *TAS4* tasiRNAs target MYB transcription factors (Rajagopalan et al., 2006). *TAS3* tasiRNAs target *ARF3* and *-4*, which are involved in developmental timing (Cho et al., 2012) and the normal development of aerial lateral organs, such as lateral roots (Marin et al., 2010; Yoon et al., 2010) and flowers (Matsui et al., 2014).

The roles of the pathway miR390-*TAS3*-*ARF3*/*-4* in root and flower development are conserved and clear in plants; however, their functions in plant embryo development are unclear. For example, miR390 peaked in mature embryos (Zhang et al., 2012), and three detected tasiRNAs generated from *TAS3*, which were triggered by miR390, peaked in mature embryos in larch (*Larix leptolepis*; Zhang et al., 2013), suggesting that the miR390-*TAS3*-tasiRNA pathway might play regulatory roles during CE development. In Valencia sweet orange, miR390 is involved in globular- shaped embryo formation (Wu et al., 2011), and over-expression of Csi- MIR390 callus lines caused lost embryogenesis capacity (Liu et al., 2013). During *Gossypium hirsutum* SE, miR390 reached the highest expression level at the EC stage, remained at moderate levels during embryo development, and one *TAS3* transcript was verified as the target of miR390 (Yang et al., 2013). As mentioned above, notably, the roles of the pathway miR390-*TAS3*-*ARF3/-4* in embryo development are somewhat confused in different plants, and their functions in plant embryos remain unclear.

*Dimocarpus longan* (longan), a member of the Sapindaceae family, is an exotic subtropical fruit mainly planted in Northern Burma, and Northeast and Southern China. Longan fruit are preferably eaten fresh because of their sweet flavor and beneficial health effects, and usually contain a relatively large black or brown seed at maturity, with a large quantity of polysaccharides (Tseng et al., 2014). The seeds have also been used as bioactive ingredients in many traditional Chinese medicines to improve human health and increase the immunomodulatory capacity (Rangkadilok et al., 2005). However, the molecular mechanism of longan seed development remains unclear because of the extreme genetic heterozygosity exhibited and the difficulty of sampling the early embryos (Liu et al., 2010; Lai and Lin, 2013; Lin and Lai, 2013a,b; Lin et al., 2015). To avoid these difficulties, longan SE, which resembles zygotic embryogenesis, has previously been used widely as a model system to investigate *in vitro* and *in vivo* regulation of embryogenesis in plants (Lai et al., 2010; Lin and Lai, 2010). Our previous work indicated that dlo-miR390 a.1 and -a^∗^.1 accumulated during heart- and TE stages (Lin and Lai, 2013b). However, how miR390 directs the formation of tasiRNAs, and down- regulates the expression of target *ARF3* and *-4* during longan SE, remains unclear.

In this study, the conserved pathway miR390-*TAS3*-*ARF3*/*-4* was identified in longan using an integrated strategy including RT-PCR/RACE, computational, genome-wide expression profiling and experimental validation. First, the primary miR390 and *TAS3* transcripts were cloned by RT-PCR and RACE. The promoter of primary miR390 was isolated by Tail-PCR and its *cis*-acting elements were predicted using bioinformatic methods. The *TAS3* tasiRNAs triggered by miR390 were detected from a longan small RNA database (Lin and Lai, 2013b). TasiRNA-ARF targets were identified by a modified RLM-RACE. The expressions of primary miR390, *DlTAS3*, *DlARF3* and *-4* were analyzed in *D. longan* ‘Honghezi’ SE and in ‘Sijimi’s tissues. Their responses to 2,4-D stimuli were also determined. These results revealed a possible role for the conserved pathway miR390- *TAS3*-*ARF3*/*-4* in longan somatic embryo development.

## Materials and Methods

### Plant Materials and Treatments

The synchronized embryogenic cultures from *D. longan* ‘Honghezi’ used in this study were friable-EC, ICpECs, GEs, TEs, and CEs; Lai and Chen, 1997). For RT-qPCR analysis, the EC were cultured on Murashige and Skoog medium (MS; Murashige and Skoog, 1962) supplemented with 1.0 mg/L 2,4-D and 2% sucrose (pH 5.8), for 20 days, then transferred to MS liquid medium containing 2,4-D (0, 0.5, 1.0, 1.5, and 2 mg/L) in a rotary shaker at 150 rpm for 24 h, respectively. All samples were collected and stored at -80°C for subsequent analyses.

Samples of roots (R), leaves (L), floral buds (FB), flowers (F), young fruits (YF), mature fruits (MF), pericarp (P1), pulp (P2), and seeds (S) collected from at least six rootstock plants of the same cultivar, *D. longan* ‘Sijimi,’ were provided by the experimental fields of the Fujian Academy of Agricultural Science in Putian, and used for RNA extraction.

### Isolation of the *miR390* Gene from *D. longan*

To gain a more complete understanding of the *miR390* gene and its evolution in plants, based on the previously published small RNA data (BioSample accession SAMN04120614, Bio-Project ID PRJNA297248; Lin and Lai, 2013b) and the longan EC transcriptome data (SRA050205; Lai and Lin, 2013), the longan primary *miR390* gene was obtained from longan EC DNA using PCR with sense (390F) and anti-sense (390R) primers. To determine the 5′-end, a GeneRacer Kit (Invitrogen, Carlsbad, CA, USA) was used for full-length cDNAs synthesis from a mixture of total RNAs (EC, ICpEC, GE, and HE), following the manufacturer’s instructions. 5′ RACE PCR was carried out using two nested PCR reactions and a combination of GeneRacer forward primers (5′ Primer and 5′ Nested Primer) and specific reverse primers (390-5RACE1 and 390-5RACE2). To obtain the gene regulatory sequence for the longan *miR390* gene, genome walking was performed to obtain flanking genomic DNA using TAIL-PCR (Takara, Japan). Its promoter was cloned by three nested PCR amplifications from a longan EC DNA template with the same forward primer, AP2, and specific reverse primers (390-SP1-SP5). The list of primers for PCR amplification is shown in **Table 1**.

**Table 1:** Primers used in pri-miR390 and *TAS3* cloning.

Primer name	Primer sequence(5′–3′)	Tm/°C	Description
390-F	CTGTGTATAGAGACATACATGATGAG	55	RT-PCR
390-R	ACCCATCAAAGATATATGATCTAGTAG		
390-5RACE1	CATGAAACTCAGGATAGATAGCGCC	55.6	5′-RACE
390-5RACE2	GTGGCGCTATCCCTGCTGAG	56.6	
390-SP1	CATGAAACTCAGGATAGATAGCGCC	55.6	Tail-PCR
390-SP2	CAACAGCTCATCATCATCATCATC	52	
390-SP3	GTGGCGCTATCCCTGCTGAG	56.6	
390-SP4	GAGCTCTCATGGTGAAGGTGG	54.8	
390-SP5	GTGGTGGAGACGGTCTTGTTG	54.8	
TAS3-F	TTCTTGACCTTGTAAGGCCTT	55	RT-PCR
TAS3-R	AGCTCAGGAGGGATAGAAG		
TAS3-3P1	TTCTTGACCTTGTAAGGCCTT	53	3′-RACE
TAS3-3P2	TTCCGTCCAACTCATCTTCTC	56	
TAS3-5P1	AAGATGAGTTGGACGGAAAC	54	5′-RACE
TAS3-5P2	TTCTTAACGCGGGATCTTAC	56	

### *MiR390*-Targeted *TAS3* Gene Cloning

In *Arabidopsis*, miR390 has been shown to target *TAS3* (Montgomery et al., 2008a), but there is no evidence for the existence of *TAS3* in *D. longan*. To verify its existence and determine the structure of the *TAS3* transcript, the conserved region of the *TAS3* transcript was first obtained using RT-PCR with sense (TAS3-F) and anti-sense (TAS3-R) primers designed from known *TAS3* sequences. To determine the 5′- and 3′-ends, 5′ and 3′ RACE PCR were carried out by nested PCR reactions using a combination of GeneRacer primers and the specific primer pairs TAS3-5P1/5P2 and TAS3-3P1/TAS3-3P2. The list of primers for PCR amplification is shown in **Table 1**.

### Bioinformatics Analysis

The *miR390* and *TAS3* genes were compared against the miRBase server (Release 21: June 2014) and NCBI using BLAST. Multiple alignment analysis and secondary structure analysis were performed using DNAMAN ver. 6.0 (Lynnon Biosoft, Pointe-Claire, QC, Canada). *Cis*-acting elements analysis was carried out using the PlantCARE database (Lescot et al., 2002). For phylogenetic analysis, MEGA 5.02 (Kumar et al., 2004) was performed according to the neighbor-joining method (Saitou and Nei, 1987) with 1000 bootstrap replicates. The psRNATarget analysis (Dai and Zhao, 2011) was carried out for miRNA and tasiRNA target prediction.

### Cleaved Target mRNA Identification with 5′ RLM-RACE

TasiRNA produced from the *TAS3* transcript has been shown to target *ARF3* and *-4* (Marin et al., 2010). To discover the target transcripts of *TAS3* tasiRNA in longan, a modified 5′ RLM-RACE experiment was set up. Here, the transcripts of *ARF3* (GenBank accession No. KJ200347.1) and *-4* (GenBank accession No. KJ200347.1) were used to designed RACE primers. The cDNA was synthesized using a GeneRacer Kit using RNA extracted from friable-EC, ICpECs, GEs, and HEs of longan embryogenic cultures. PCR amplification of cDNA fragments using 5′ RACE outer primers and gene-specific reverse primers was performed. PCR fragments were cloned and sequenced to identify the 5′-end of the amplified target genes.

### RNA Extraction and RT-qPCR

For RT-qPCR analysis, total RNAs were extracted from the above-mentioned samples using a TRIzol Reagent kit (Life Technologies, Grand Island, NY, USA). cDNA for mRNA quantification was subsequently synthesized using PrimeScript ^TM^ RT Reagent Kit Perfect Real Time (Takara Code, DR037A, Japan). For RT-qPCR amplification, cDNA was diluted 15 times before use. RT-qPCR was performed using LightCycler 480 equipment (Roche Applied Science, Switzerland) using the manufacturer’s instructions. Three reference genes *DlFSD1a*, *EF-1a*, and *eIF-4a* (Lin and Lai, 2010) were used to normalize our signal. All reactions were performed in triplicate. Statistical analysis was performed using SPSS 19. The gene names and primer sequences are provided in **Table 2**.

**Table 2:** Real-time quantitative reverse transcription PCR (RT-qPCR) primers.

Primer name	Primer sequence 5′–3′)	Product size/bp	Tm/°C
miR390	TCGCTATCCATCCTGAGTTTC	—	60
pri-miR390-QF	GATGATGATGATGATGAGCT	125	56
pri-miR390-QR	GCCTAGAAGAGACAAGTCGTT		
TAS3-QF	TCCATCGTCAAAAACTAGAAGG	123	56
TAS3-QR	ATGCTTGTGTCCTCCTTCATAC		
ARF3-QF	AGATTCCGACACATCTACCG	189	58
ARF3-QR	AGGTGGAAATGTAGCAGCAC		
ARF4-QF	TCAAGATCCCACAATGCG	101	58
ARF4-QR	TAACTTCATCCCCAACAGCC		

## Results

### Cloning and Analysis of the *D. longan miR390* Gene Sequence

**FIGURE 1:**
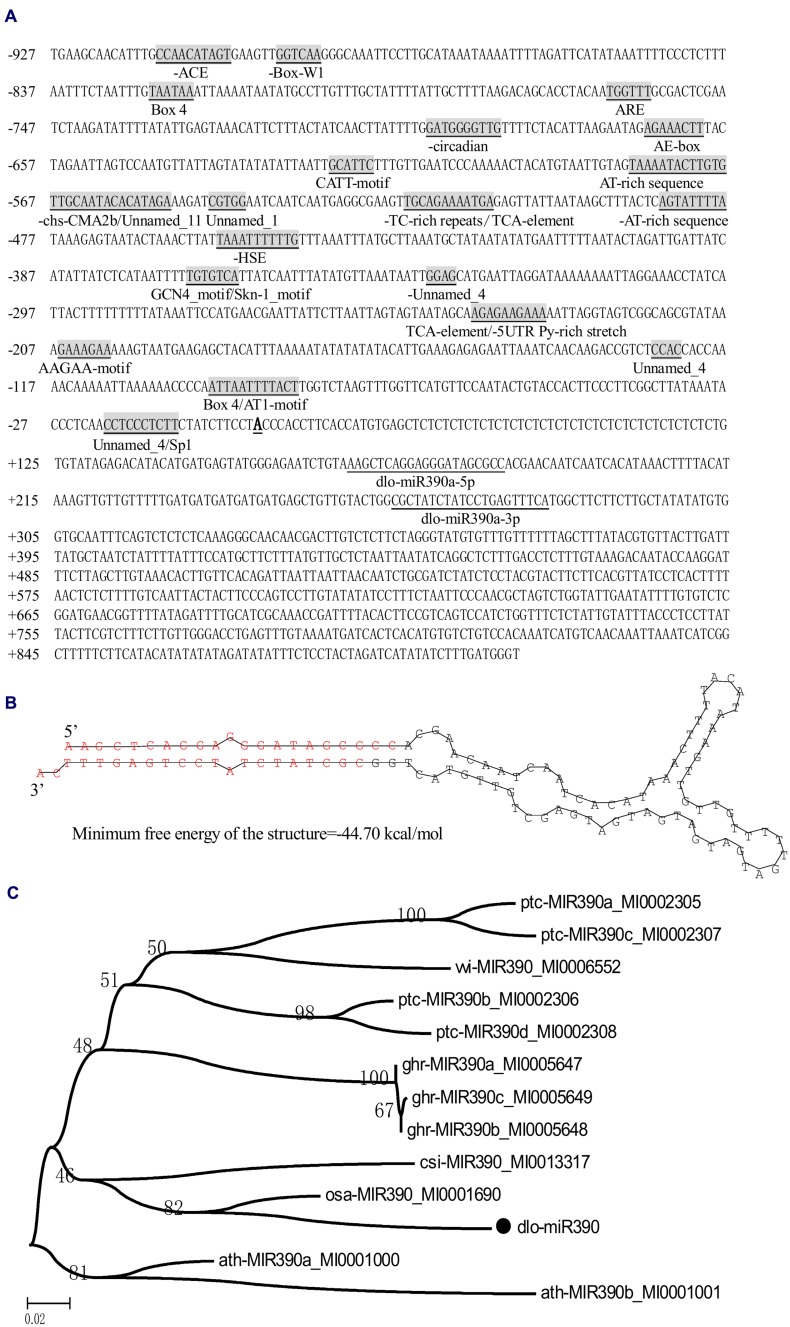
**MiR390 transcript in *D. longan*. (A)** The primary miR390 sequence and its 5′ flanking sequence. Mature miR390 sequences are underlined, and the names are given under the sequences. The transcription start site (TSS) is *Bold* and *underlined*; *cis*-acting regulatory elements are *underlined*, gray shaded, and the names are given under the elements. **(B)** Hairpin structure of the longan miR390 precursor. **(C)** Bootstrapped neighbor joining phylogenetic tree of precursor miR390 in plant species. Bootstrap values are in percentages. dlo, *Dimocarpus longan*; ptc, *Populus trichocarpa*; ghr, *Gossypium hirsutum*; vvi*, Vitis vinifera;* ath, *Arabidopsis thaliana*; osa, *Oryza sativa*; *cis*, *Citrus sinensis.*

To determine miR390s structure and the length of the gene in the *D. longan* genome, the full-length cDNA sequence of the primary miR390 transcript was obtained by RT-PCR and 5′-RACE. According to previous studies, the longan mature miR390 (Lin and Lai, 2013b) was predicted to derive from the transcript Contig648826, which was retrieved from longan EC transcriptome data (SRA050205; Lai and Lin, 2013). To verify the existence of the transcript, it was cloned from longan EC using RT-PCR and 5′ RACE. Sequence analysis showed that the transcript was 845 bp in length and had no introns (GenBank accession No. KJ372216), and the first nucleotide (transcription initiation site, TTS) of the full-length cDNA was A (**Figure 1A**); the mature sequences dlo-miR390a-5p and dlo-miR390-3p were found to exactly match the 5′ arms of the transcript (**Figure 1A**). A sequence of 117 bp extracted from between the positions of dlo-miR390-5p and -390-3p in the transcript was further used to predict the secondary structure using DNAMan6.0 software. The analysis showed that the sequence could form the classic stem-loop structure and the minimum free energy of the structure was -44.7 kcal/mole (**Figure 1B**). The sequence has similarities with existing precursor miR390s in *Malus domestica*, *G. hirsutum*, *Glycine max*, and *Cucumis melo* using BLAST analysis, and is phylogenetically closely related to pre-miR390 transcripts of *Arabidopsis lyrata*, *Populus trichocarpa*, and *Vitis vinifera*, but was distant from pre-miR390s of *C. melo*, *M. domestica*, and *Prunus persica* (**Figure 1C**).

### Isolation of the Longan miR390 Promoter and Analysis of its Structural Features

Promoter analysis is an essential step in the identification of regulatory networks. Here, the partial 5′-flanking region of miR390 (927 bp) upstream of the TTS was isolated from longan friable-EC genomic DNA (GenBank accession No. KJ372219), and a promoter motif search was performed by PlantCARE (Lescot et al., 2002). The analysis indicated that the classical promoter elements, such as a TATA box and a number of CAAT boxes, were located in the expected position of the miR390 promoter (i.e., within -30 to -90 nucleotides upstream of the TTS). Potential *cis*-regulatory elements associated with light, including an ACE motif, an AE-box, an AT1-motif, three Box 4 sites, a CATT-motif, an Sp1 motif and a chs-CMA2b motif, and the *cis*-acting elements related to stress-related responses containing a HSE motif (involved in heat stress responses) and TC-rich repeats (involved in defense and stress responses), were also detected in the promoter region. In addition, a TCA-element involved in SA responsiveness; an ARE *cis*-acting regulatory element that is essential for anaerobic induction; two AT-rich sequence elements for maximal elicitor-mediated activation (two copies); a Box-W1 element involved in fungal elicitor responsiveness; a Skn-1 motif and a GCN4 motif, which are involved in endosperm expression; a circadian element involved in circadian control; a *cis*-acting element that confers high transcription levels (5′ UTR Py-rich stretch); and four elements of unknown function were also present in the promoter (**Figure 1A**). These results indicated that the *miR390* gene could be regulated by environmental (light and stress) factors and hormones (SA).

### Cloning and Analysis of miR390-Targeted *TAS3* Gene in Longan

**FIGURE 2:**
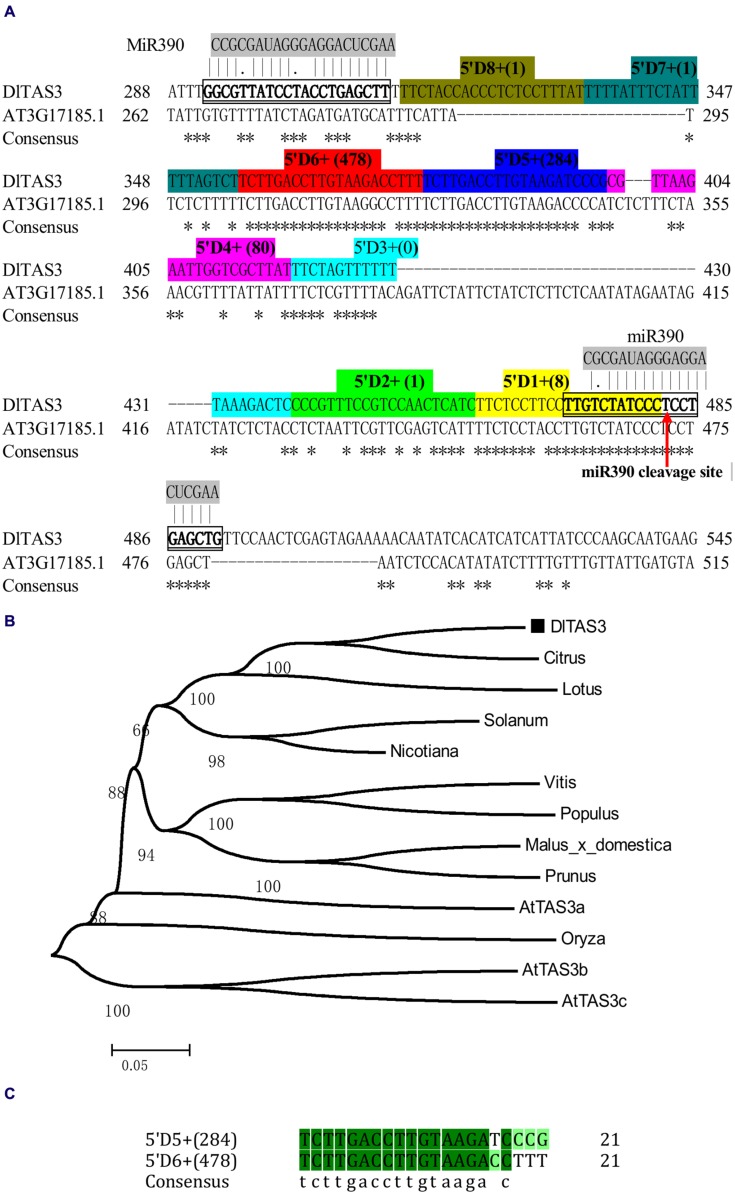
**Longan *TAS3* transcript. (A)** Sequence alignment of *DlTAS3* in *D. longan* and AT3G17185.1 (*TAS3*) in *A. thaliana*. Asterisk represents the consensus nucleotide; *TAS3*- tasiRNAs named as 5’D1+ (reads), 5’D2+ (reads), and so on, are highlighted in different colors. The miR390 cleavage site in *TAS3* transcript is showed by red arrow. **(B)** Phylogenetic relationships among plant *TAS3* sequences using the neighbor-joining method. Bootstrap values are in percentages. GenBank accession numbers for each sequence represented in the tree are as follows: *A. thaliana* (AtTAS3a, At3g17185; AtTAS3b, At5g49615; AtTAS3c, At5g57735), *V. vinifera* (FQ386573); *Lotus japonicus* (AK338955); *Malus domestica* (XR_524192); *C. sinensis* (XR_371831); *Solanum lycopersicum* (JX047545); *O. barthii* (GQ420228); *Nicotiana tabacum* (FJ804751); *Prunus mume* (XR_513520); *P. trichocarpa* (XM_006378492). **(C)** Sequence alignment between 5’D5+ (284) and 5’D6+ (478).

In this study, based on the sequences of *TAS3* in other plants, the full-length cDNA of *TAS3* was obtained through RT-PCR and 5′/3′ RACE using longan EC cDNA as the template. Sequence analysis showed that the *TAS3* transcript, named *DlTAS3*, comprised 579 bp, with a 23-bp poly A tail (GenBank accession No. KJ372220). Sequence alignment analysis indicated that *DlTAS3* has high similarities with *Arabidopsis TAS3* (AT3G17185.1). The mature miRNA sequence, dlo-miR390a-5p, was predicted to bind exactly to the *DlTAS3* transcript at two sites, located at positions 292–312 and 472–491 bp, respectively (**Figure 2A**). The phylogenetic analysis (**Figure 2B**) suggested that *DlTAS3* in *D. longan* is closely related to *TAS3* in *Citrus sinensis*, *Lotus japonicus*, and *Solanum lycopersicum*, but was distant from *TAS3* in *A. thaliana* and *Oryza barthii.*

### MiR390 Guides In-phase Processing of *DlTAS3* Transcripts

*TAS3* tasiRNAs originate from sequences between the two miR390 target sites (Kikuchi et al., 2006; Montgomery et al., 2008a). Here, the sequence of *DlTAS3* between the two binding sites was 158 bp, and the dlo-miR390-guided cleavage site was predicted to be located near the 3′ end of the *DlTAS3* transcript, while dlo-miR390 interacts in a non-cleavage mode with a second site near the 5′ end, which was found to have a conserved mismatch at the center of the binding region (**Figure 2A**).

**FIGURE 3:**
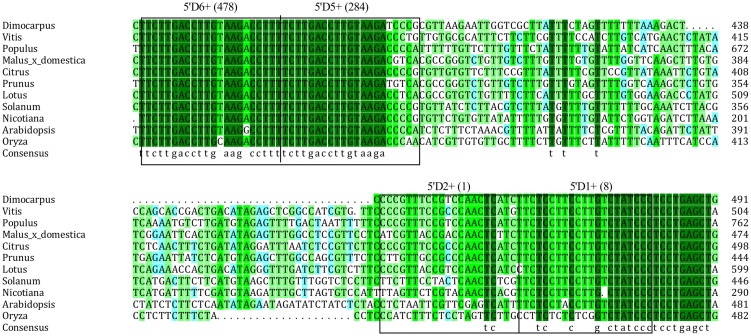
**Alignment of *TAS3* sequences corresponding to the *TAS3* tasiRNAs in orthologs of 11 species**. GenBank accession numbers are as follows: *V. vinifera* (FQ386573), *P. trichocarpa* (XM_006378492), *M. domestica* (XR_ 524192), *C. sinensis* (XR_371831), *Prunus mume* (XR_513520), *S. lycopersicum* (JX047545), *L. japonicus* (AK338955), *Nicotiana tabacum* (FJ804751), *A. thaliana* (At3g17185), *O. barthii* (GQ420228).

MicroRNAs direct tasiRNA biogenesis in plants, and miR390-guided cleavage was shown to set the 21-nucleotides phase for tasiRNA precursor processing (Kikuchi et al., 2006). Here, according to the dlo-miR390-guided cleavage site, eight potential tasiRNAs with the 21-nucleotides phase were predicted to be produced from miR390-guided *DlTAS3* cleavage (**Figure 2A**; **Table 3**). In-phase, the 21-nucleotides positions on the 5′ side of the miR390 cleavage site were named 5′D1+, 5′D2+, and so on. Among these tasiRNAs, the TAS3_ 5′D5+ and 5′D6+ have a high similarity, with only four nucleotides differences at the 3′ end of the tasiRNAs (**Figure 2C**), and they are also very similar to the 5′D7+ and 5′D8+ of the *TAS3* in *A. thaliana* (Kikuchi et al., 2006), *G. max* (Hu et al., 2013), *M. domestica*, *C. sinensis*, and *P. trichocarpa* (**Figure 3**). These results suggested that miR390- directed *TAS3* cleavage leading to the production of tasiRNA is conserved in plants.

**Table 3:** The sequence abundance of longan *TAS3* tasiRNA.

No.	tasiRNA sequence and color	Reads in smallRNA database
D1+	TTCTCCTTCCTTGTCTATCCC	8
D2+	CCCGTTTCCGTCCAACTCATC	1
D2-	CTACTCAACCTGCCTTTGCCC	26
D3+	TTCTAGTTTTTTTAAAGACTC	No hits found
D4+	CGTTAAGAATTGGTCGCTTAT	80
D5+	TCTTGACCTTGTAAGATCCCG	284
D6+	TCTTGACCTTGTAAGACCTTT	478
D7+	TTTTATTTCTATTTTTAGTCT	1
D8+	TTCTACCACCCTCTCCTTTAT	1

### Longan tasiRNA Abundance Analysis and their Target Prediction

To verify the existence of *DlTAS3* tasiRNAs in longan, the tasiRNAs predicted in Section “MiR390 Guides In-phase Processing of *DlTAS3* Transcripts” were searched for in a longan small RNA dataset using local BLAST (Lin and Lai, 2013b). Among the eight tasiRNAs, the reads of TAS3_5′D5+ and 5′D6+ siRNAs were more abundant, with 284 and 478 reads. This result was similar to previous findings in which the reads of *TAS3* 5′D7+/5′D8+ tasiRNAs were also high in *G. max* (Hu et al., 2013). The signatures of 5′D2- and 5′D4+ siRNAs were 14 and 44 reads, respectively, while the expressions of other tasiRNAs were very low, and the 5′D3+ siRNA was not detected in longan SE (**Figure 2A**). These results further proved that dlo-miR390 was sufficient to trigger secondary tasiRNA biogenesis, and miR390-guided *TAS3* cleavage with the 21-nucleotides phase was conserved in plants.

TasiRNAs interact with target homologous mRNAs and guide cleavage by the same mechanism as plant miRNAs (Hao et al., 2006; Ruduś et al., 2006). Here, to identify the targets of *DlTAS3*_tasiRNAs, the three with the highest abundances, TAS3_5′D4+, 5′D5+, and 5′D6+ siRNAs, were matched against nucleic acid sequences of the longan *ARF* gene families and the transcripts library removed the miRNA gene of *A. thaliana* (TAIR, version 10, released on 2010.12.14). The psRNATarget analysis indicated that 10 mRNAs were predicted to be targets of TAS3_5′D4+, 5′D5+ and 5′D6+ (**Table 4**). The TAS3_5′D4+ targets RNA-dependent RNA polymerase 6 (RDR6) and TRS120 mRNAs for cleavage. The 5′D5+ and 5′D6+ tasiRNAs both target *ARF3/-4* from *A. thaliana* or *D. longan*, indicating that these tasiRNA binding sites are highly conserved among different plants. In addition, *DlARF3* and *-4* contained two complementary sites to the *TAS3*_5′D5+/5′D6+ tasiRNAs, which result was consistent with previous studies in *A. thaliana* (Kikuchi et al., 2006) and *G. max* (Hu et al., 2013). Moreover, TAS3_5′D5+ also targets PXA1 (peroxisomal ABC transporter 1), NAC (No Apical Meristem, domain transcriptional regulator super family protein), SPL5 (squamosa promoter binding protein-like 5), and UPL2 (ubiquitin-protein ligase 2); and TAS3_5′D6+ also targets MEI1 (transcription coactivators), Core-2/I-branching beta-1, and 6-*N*-acetylglucosaminyltransferase family protein. These results suggested that tasiRNAs are broadly involved in plant development by guiding the cleavage of different targets, similar to plant miRNAs.

**Table 4:** Identified potential target genes of longan *TAS3*-tasiRNA.

tasiRNA	Target accession No.	Target description	Target_end
D4+	AT5G11040	RDR6, RNA-dependent RNA polymerase 6	miRNA 21 UAUUCGCUGGUUAAGAAUUGC 1 Target 1284 AUAAACGACCAGUUUUUGAUG 1304
	AT5G11040	TRS120	miRNA 20 AUUCGCUGGUUAAGAAUUGC 1 Target 2904 UAAGCUACCGGUUCUUGAUG 2923
D5+	AT2G33860	ARF3, Transcriptional factor B3 family protein / auxinresponsive factor AUX/ IAA- relate	miRNA 20 CCCUAGAAUGUUCCAGUUCU 1 Target 1674 AGGGUCUUGCAAGGUCAAGA 1693
	KJ200347	ARF3, auxin-responsive factor AUX/IAA- relate	miRNA 20 CCCUAGAAUGUUCCAGUUCU 1 Target 1470 AAGGUCUUGCAAGGUCAAGA 1489 miRNA 20 CCCUAGAAUGUUCCAGUUCU 1 Target 1680 AAGGUCUUGCAAGGUCAAGA 1699
	AT5G60450	ARF4, auxin response factor 4	miRNA 20 CCCUAGAAUGUUCCAGUUCU 1 Target 2084 AGGGUCUUGCAAGGUCAAGA 2103
	KJ200347	ARF4, auxin response factor 4	miRNA 20 CCCUAGAAUGUUCCAGUUCU 1 Target 1462 AAGGUCUUGCAAGGUCAAGA 1481 miRNA 20 CCCUAGAAUGUUCCAGUUCU 1 Target 1663 AAGGUCUUGCAAGGUCAAGA 1682
	AT3G12910	NAC (no apical meristem) domain transcriptional regulator superfamily protein	miRNA 21 GCCCUAGAAUGUUCCAGUUCU 1 Target 238 UGGGAUCUUCCAAGGUCGAGG 258
	AT4G39850	Peroxisomal ABC transporter 1	miRNA 21 GCCCUAGAAUGUUCCAGUUCU 1 Target 374 CGGGGUCUUGUAGCGUCAAGA 394
	AT3G15270	SPL5, squamosa promoter binding protein-like 5	miRNA 21 GCCCUAGAAUGUUCCAGUUCU 1 Target 704 UGGCAUCUAACAAUGUCAAGA 724
	AT1G70320	UPL2 | ubiquitin-protein ligase 2	miRNA 21 GCCCUAGAAUGUUCCAGUUCU 1 Target 3848 UGGGAUUUU-CAAGGUCAAGG 3867
D6+	AT2G33860	ARF3, transcriptional factor B3 family protein/auxinresponsive factor AUX/IAA- relate	miRNA 20 UUCCAGAAUGUUCCAGUUCU 1 Target 1794 AAGGUCUUGCAAGGUCAAGA 1813
	KJ200347	ARF3, transcriptional factor B3 family protein/auxinresponsive factor AUX/ IAA- relate	miRNA 21 UUUCCAGAAUGUUCCAGUUCU 1 Target 1469 GAAGGUCUUGCAAGGUCAAGA 1489 miRNA 20 UUCCAGAAUGUUCCAGUUCU 1 Target 1680 AAGGUCUUGCAAGGUCAAGA 1699
	AT5G60450	ARF4, auxin response factor 4	miRNA 21 UUUCCAGAAUGUUCCAGUUCU 1 Target 2083 AAGGGUCUUGCAAGGUCAAGA 2103
	KJ200347	ARF4, auxin response factor 4	miRNA 21 UUUCCAGAAUGUUCCAGUUCU 1 Target 1662 GAAGGUCUUGCAAGGUCAAGA 1682 miRNA 20 UUCCAGAAUGUUCCAGUUCU 1 Target 1462 AAGGUCUUGCAAGGUCAAGA 1481
	AT1G77320	MEI1, transcription coactivators	miRNA 21 UUUCCAGAAUGUUCCAGUUCU 1 Target 66 GAAGCUCUUACAAAGUCGAGA 86
	AT5G57270	Core-2/I-branching beta-1,6- *N*acetylglucosaminyltransferase family protein	miRNA 20 UUCCAGAAUGUUCCAGUUCU 1 Target 516 AAGUUUUUCCAGGGUCAAGA 535

### Validation of tasiRNA-Guided Cleavage of Target Gene *DlARF3* and *-4* mRNAs in Longan

**FIGURE 4:**
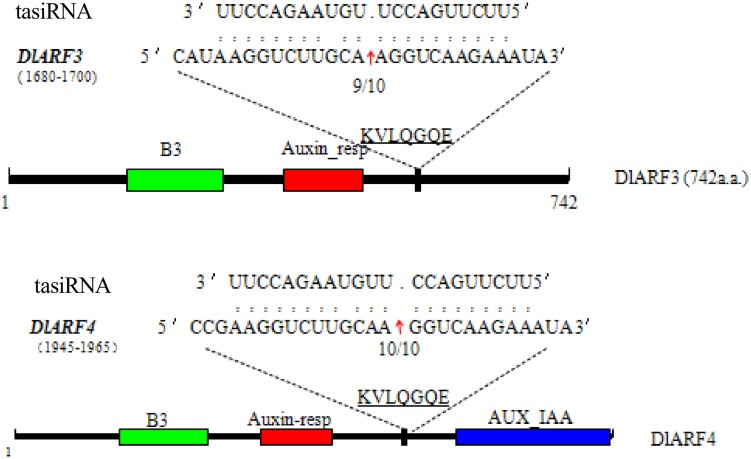
**Validation of tasiRNA guided cleavage of target genes *DlARF3*, *-4* and mapping of the cleavage sites in target gene mRNAs**. Numbers indicate the fraction of cloned PCR products. The B3 DNA binding domain (B3), Auxin_resp, and AUX_IAA, are highlighted in green, red, and blue in the ARF3 and -4 proteins, respectively. The tasiRNA complementary sequence in the *DlARF3* and *-4* mRNA and the corresponding amino acid sequences are shown.

Four *ARF* genes have been validated as the target of TAS3 5_D7+/5_D8+ siRNAs in *Arabidopsis* (Kumria et al., 2003). In our study, only *DlARF3* and *-4* were predicted to be targets of *TAS3*_5′D5+/5′D6+ tasiRNAs, which are similar to TAS3 5_D7+/5_D8+ siRNAs in *Arabidopsis*. Therefore, to further verify the cleavage of *DlARF3* and *-4* by the *TAS3*_5′D5+/5′D6+ tasiRNAs, a modified RLM-RACE analysis was performed, which resulted in the detection of fragments of *DlARF3* and *-4* mRNAs from longan embryogenic tissues (**Figure 4**). Fragment sequence analysis showed that the cleavage sites in *DlARF3* and *-4* mRNAs are located at positions corresponding to the amino acid sequences KVLQGQE; in addition, *DlARF3* was cleaved between the 10th and 11th nucleotides complementary to miR390, while *DlARF4* was cleaved between the 9th and 10th nucleotides of miR390 (**Figure 4**). These results clearly indicated that *TAS3*_5′D5+/5′D6+ tasiRNAs cleaved the *DlARF3* and *-4* mRNAs during longan SE, which further proved that the tasiRNA-directed *ARF* mRNA cleavage was conserved in plants.

### Expression Profiling of miR390-*DlTAS3*-*DlARF3/-4* during Longan SE

**FIGURE 5:**
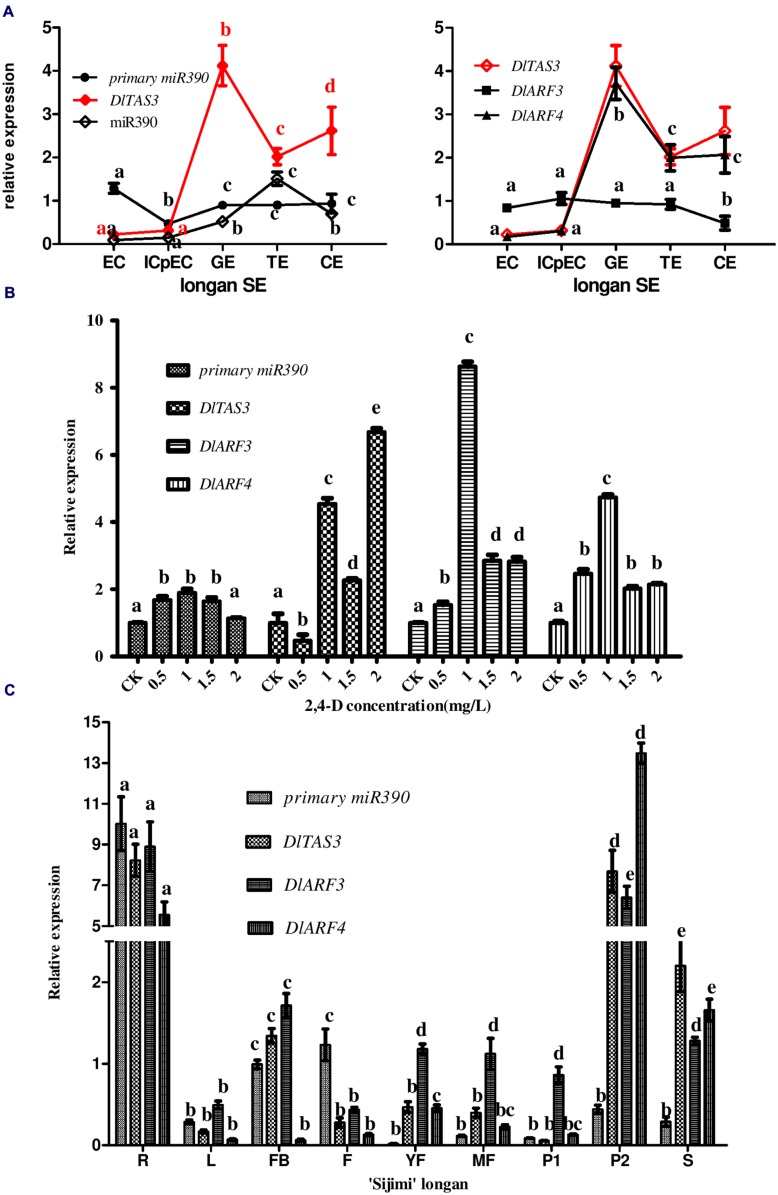
**Expression profiles of miR390, pri-miR390, *DlTAS3*, *DlARF3* and *-4* in *D. longan*. (A)** Relative expressions of miR390, pri-miR390, *DlTAS3*, *DlARF3* and *-4* during longan SE. Samples: EC, friable-embryogenic callus; ICpEC, incomplete compact pro-embryogenic cultures; GE, globular embryos; TE, torpedo-shaped embryos; CE, cotyledonary embryos. Expression level was normalized to the reference genes *DlFSD1a*, *EF-1a*, and *eIF-4a*. **(B)** Auxin control of primary miR390, *DlTAS3*, *DlARF3* and *-4* expression levels. The expression levels were normalized to the reference gene *EF-1a*. **(C)** Differential expression analysis of primary miR390, *DlTAS3*, *DlARF3* and *-4* in vegetative and reproductive tissues of ‘Sijimi’ longan. Samples: R, roots; L, leaves; FB, floral buds; F, flowers; YF, young fruit; MF, mature fruit; P1, pericarp; P2, pulp; S, seeds. The expression level was normalized to the reference genes *DlFSD1a*, *EF-1a*, and *eIF-4a*; The y-axes represent the relative expression values; the x-axes represent the vegetative and reproductive samples of ‘Sijimi’ longan. Different lowercase letters above the bars indicate a statistically significant difference, and identical lowercase letters denote no significant difference among different samples (*P* < 0.05).

To systematically analyze the function of miR390-*TAS3*-*ARF3/-4* during longan SE, expression profiling was performed using five embryogenic cultures. RT-qPCR analysis showed that the pri-miR*390*, miR390, *DlTAS3*, *DlARF3* and *-4* exhibited different temporal and spatial expressions (**Figure 5A**). Pri-miR390 was highly expressed in EC, and less expressed in ICpECs, while it had a stable expression level with no fluctuations from GEs to CEs, suggesting that the accumulated pri-miR390 in the EC stage may be necessary for the maintenance of embryonic callus in an undifferentiated state in longan. miR390 showed its lowest expression in EC and highest expression in TEs, with a general lack of correlation of pri-miRNAs at the transcription level. It is worth noting that *DlTAS3* and *-4* showed similar expression patterns. They both exhibited their lowest expressions in EC, and reached their peaks in the GEs stage, which were mainly inversely proportional to the expression of miR390, especially at the GEs to CEs stages. *DlARF3* showed little variation from the EC to TEs stages, and exhibited its lowest expression in the CEs stage. There was a general lack of correlation between the expressions of *DlARF3* and *TAS3*. We concluded that miR390 down-regulation of *TAS3* leading to the production of tasiRNAs via cleavage of *DlARF3/-4* mRNAs during somatic embryo development in longan.

### MiR390-*DlTAS3*-*DlARF3/-4* Expressions in Response to Auxin in Longan EC

A previous study showed that miR390 and tasiRNA ARF are regulated by auxin concentration (IAA; Yoon et al., 2010). Embryogenesis is inhibited by exogenously supplied 2,4-D (>10^-9^ M) or indoleacetic acid (IAA; >10^-10^ M) in plants (Ruduś et al., 2002). Here, to examine how the pathway of miR390-*TAS3*- *ARF3/-4* expression is regulated by 2,4-D, their expression levels were monitored in longan EC exposed to different 2,4-D concentrations (**Figure 5B**). The results showed that their levels all accumulated at 1.0 mg/L 2,4-D compared with auxin-free medium under the same conditions, but decreased at higher concentrations, except for *DlTAS3*. In contrast to miR390-*ARF3/-4*, *DlTAS3* was expressed at its lowest level at 0.5 mg/L 2,4-D compared with auxin-free, increased at 1.0 mg/L, but decreased at 1.5 mg/L, and reached its peak at 2.0 mg/L (**Figure 5B**). This result demonstrated that the presence of auxin in the medium controlled the level of miR390-*TAS3*-*ARF3/-4*, which is similar to the results of a previous study (Yoon et al., 2010).

### MiR390-*DlTAS3*-*DlARF3/-4* Expressions in ‘Sijimi’ Longan Tissue Types

To more precisely pinpoint the locations of miR390-*TAS3-ARF*3/-4 expressions, the tissues of the cultivar ‘Sijimi’ longan at nine stages of development, roots (R), leaves (L), floral buds (FB), flowers (F), young fruits (YF), mature fruits (MF), pericarp (P1), pulp (P2), and seeds (S) were used to assay RNA accumulation.

The results showed that primary transcripts of miR390, *DlTAS3*, *DlARF3* and *-4* exhibited more or less expression in vegetative and reproductive organs, and were preferentially expressed in roots (**Figure 5C**), suggesting they might affect ‘Sijimi’ root development. In addition, *DlTAS3*, *DlARF3* and *-4* were also expressed at relatively high levels in pulp (P2) and seeds (S), while the primary miR390 transcript expression was an exception, implying that *TAS3-ARF3/-4* may also be involved in the fruit development of longan. Furthermore, the expressions of pri-miR390 in young fruits (YF), mature fruits (MF), and pericarp (P1), *DlTAS3* in pericarp (P1), and *DlARF4* in leaves (L) and flower buds (FB) in mature fruits were low. These data revealed extensive regulation roles of miR390-*TAS3*-*ARF3/-4* signal in the developmental stages of vegetative and reproductive growth in ‘Sijimi’ longan.

## Discussion

MiRNAs and tasiRNAs are small RNAs of ∼21 nucleotides in length that have various roles as negative regulators of mRNA targets in plant physiology and development (Chuck et al., 2009). TasiRNAs originate from *TAS3* families through miR390-guided initiation-cleavage of primary transcripts and target *ARF2/-3/-4*, which are involved in the normal development of lateral roots (Marin et al., 2010;Yoon et al., 2010) and flowers in plants (Matsui et al., 2014); however, their roles in embryo development are still unclear. Here, we cloned, identified and examined the expressions of primary miR390 and its promoter, *DlTAS3*, tasiRNA, and its targets *DlARF3/-4* in longan. This study was the first systematic investigation of the conserved pathway miR390-*TAS3*- *ARF3*/*-4* in longan SE, thereby providing insights into a possible role in plant somatic embryo development.

### Characteristics of Longan Primary miR390 and its Promoter

MicroRNAs and tasiRNAs are both produced from larger capped and polyadenylated precursor non-coding transcripts of a few hundred base pairs in size (Krasnikova et al., 2009). However, the precursors of miRNAs or tasiRNAs are little studied and cloned, and have only been reported in some genome-sequenced or model plants, such as *Arabidopsis* (Xie et al., 2005) and *G. max* (Han et al., 2014). In non-genome- sequenced or non-model plants, it is hard to clone the precursors of small RNA. Here, based on the transcriptome data and RT-PCR/RACE, we successfully cloned the primary transcripts of miR390 and *TAS3*, and identified their TTS without genome data. These results laid the foundation for further experiments to identify the functions of longan miR390 and *TAS3*.

MiR390 has two genomic loci, on chromosome 2 (*MIR390a*) and 5 (*MIR390b*) in *Arabidopsis* (Montgomery et al., 2008a); the mature miR390 mostly originates from the *MIR390a* and not from the *MIR390b* locus in roots (Marin et al., 2010). *MIR390a* exhibited higher processing accuracy and efficiency of miR390 than the *MIR390b* (Cuperus et al., 2010). In longan, only one primary miR390 was cloned and its precursor was closest to pre-miR390s identified from *C. sinensis* and *O. sativa*, but closer to pre-miR390a and close to pre-miR390b in *Arabidopsis*, suggesting that the longan pre-miR390 cloned in our study perhaps has a similar function to the *Arabidopsis MIR390a*.

Previously, miR390, which has been reported to respond to UV-B in *Populus tremula* (Jia et al., 2009), Cd (Ding et al., 2011) and As (Srivastava et al., 2013) stress in rice, was down-regulated in response to Al (Chen et al., 2012) and Hg-toxicity (Zhou et al., 2012) in *Medicago truncatula*, and up-regulated under drought stress in *Vigna unguiculata* (Barrera-Figueroa et al., 2011) and *Brachypodium distachyon* (Budak and Akpinar, 2011). In our study, the *cis*-acting elements of miR390 promoter related to TC-rich repeats (defense and stress responses), and a Box-W1 element, which is a binding site for the WRKY transcriptional regulators that control pathogen defense, wound response, and senescence (Eulgem et al., 2000), were also identified, indicating the potential role of miR390 in stress responses in longan. In addition, miR390 was differentially regulated under heat conditions in switchgrass (Hivrale et al., 2015) and *Brassica rapa* (Yu et al., 2012). In longan, a *cis*-acting element related to heat stress responses (HSE motif) was identified, which was also present in miR390 promoter in strawberry (Li et al., 2014), indicating a potential role for heat in the control of miR390 accumulation in plants.

Previous studies showed that miR390 and tasiRNA ARF were positively regulated by auxin (IAA) concentration, but were not sensitive to abscisic acid, gibberellins, and cytokinin (Yoon et al., 2010; Li et al., 2013). Here, although no auxin-related *cis*-elements were identified in the longan miR390 promoter, pri-miR390 was up-regulated by 2,4-D treatment, suggesting that ARF elements may be present outside of the 927 bp promoter region; in addition, *DlTAS3*, *DlARF3* and *-4* expressions were also up-regulated by 2,4-D in a concentration-dependent manner, implying that 2,4-D may influence the activity of the *tasiRNA-ARF* pathway through its concentration-dependent regulation of *miR390* expression. However, the negative regulatory relationship was not observed among *miR390*, *DlTAS3*, *DlARF3* and *-4* under 2,4-D treatment, suggesting that the molecular mechanism of these genes response to 2,4-D is more complicated than expected, this needs further experimental verify. It is worth noting that the primary *miR390*, whose promoter contains a SA- responsive element (TCA element), was not sensitive to SA treatment (50, 75, and 100 mg/L) compared with hormone free (data not shown). A previous study showed that the mature miR390s response to auxin was significantly broader than that of the miR390 promoter GUS expression (Yoon et al., 2010), leading to a possible interpretation that SA affects the expression of mature miR390 but does not affect the expression of primary miR390 in longan.

### *TAS3* and tasiRNA Expressed in Longan SE

MiR390 targets *TAS3*. Here, a 579-bp cDNA was cloned from longan EC cDNA, which was homologous to *TAS3* in plants. The data presented here provide further evidence for the existence of *TAS3* in *D. longan*. Three *TAS3* loci have been identified in *Arabidopsis*: TAS3a (At3g17185), TAS3b (At5g49615), and TAS3c (At5g57735; Howell et al., 2007). In our study, the *DlTAS3* is phylogenetically closer to *AtTAS3a* than *AtTAS3b/c*, suggesting that *DlTAS3* may have a similar function to *AtTAS3a*. *TAS3* tasiRNAs arise from the *TAS3* family, all members of which have conserved two miR390-guided target sites; for each member, the 3′ miR390 target site, but not the 5′ target site, was cleaved (Howell et al., 2007). Recently, however, the 5′ miR390 binding site in *TAS* transcripts was demonstrated to undergo cleavage for the initiation of processing in dicots (Krasnikova et al., 2009). In this study, two miR390 target sites also existed in *DlTAS3*, and the cleaved site was predicted to be located near the 3′ end of the *DlTAS3*. Moreover, the longan small RNA data analysis showed that tasiRNAs of 21 nucleotides formed by miR390-guided cleavage on the 3′ side of *DlTAS3* were also found in longan SE, which further proved that the roles of miR390-cleaved *TAS3* in the production of tasiRNAs are also functional and conserved in longan.

In the longan small RNA data, eight *DlTAS3* tasiRNAs were found, and the reads of TAS3_5′D5+ and 5′D6+ tasiRNAs, which are similar to the tasiRNAs of *TAS3* 5′D7+/5′D8+ identified from *G. max* (Hu et al., 2013) and *L. leptolepis* (Zhang et al., 2013), were more abundant during longan SE, suggesting that their roles might be related to the development of longan SE. TasiRNAs guide homologous mRNAs cleavage, as do plant miRNAs (Hao et al., 2006; Ruduś et al., 2006). Plants *TAS3* 5′D7+/5′D8+ both target *ARF3* and *-4* (Kikuchi et al., 2006). The longan 5′D5+ and 5′D6+ tasiRNAs, which target *ARF*3 and *-4*, were also verified, suggesting that the miR390-tasiRNA-*ARF3/-4* pathway is conserved in different plants. It is worth mentioning that the longan TAS3_5′ D4+ targets RNA-dependent RNA polymerase 6 (RDR6), which is required for the production of tasiRNAs in *Arabidopsis* (Hao et al., 2006), suggesting the existence of an interactive relationship between tasiRNA and RDR6 during longan SE.

### MiR390, *DlTAS3*, *DlARF3* and *-4* Define an Auto-regulatory Network in Longan Somatic Embryo Development

We established that the pathway miR390-TAS3-*ARF3/-4* operates in longan, like other plants, but its roles during plant SE remained unclear. In our study, primary miR390 reached a peak in EC, which is consistent with the mature miR390 expression in *G. hirsutum* (Yang et al., 2013). The mature miR390 showed its lowest expression in EC and highest expression in TEs in longan, and reached a peak in globular-shaped embryo in Valencia sweet orange (Wu et al., 2011). This implied that miR390 has different expression patterns during SE, suggesting that miR390 has different roles during SE in different plants. In addition, there was a general lack of correlation between the expression of pri-miRNAs and the corresponding mature miR390s in longan, which is a common phenomenon in eukaryotes (Lee et al., 2008), suggesting that unknown mechanisms that control processing play a critical role in regulating the mature miRNA expression.

Previous studies showed that the miR390-*TAS3* tasiRNA pathway might play regulatory roles in the development of mature embryos in larch (Zhang et al., 2012, 2013). Here, *DlTAS3* exhibited the lowest expressions in EC, and reached its peaks in the GEs stage, which was mostly the reverse of the expression of miR390, further confirming that miR390 cleaved *DlTAS3*, leading to the production of tasiRNAs in longan. A previous study observed that the expression pattern of TAS3_5′D7+ was identical to that of miR390 during SE in *B. napus* (Zhao et al., 2012), suggesting that the expression of *TAS3* tasiRNA was induced by miR390. In addition, *TAS3* mRNA reached its peak at EC after 1–5 days of sub-culture in larch (Zhang et al., 2012), which was distinct from that of longan SE, implying that the roles of the miR390-*TAS3* signal are not conserved between angiosperm and gymnosperm species. Moreover, parallel expression of *DlTAS3* and *DlARF4* during longan SE was observed, while *DlARF3* showed little variation among the tissues. Obviously, the expression levels between *DlTAS3* and *DlARF3* were largely not correlated at the transcript levels, which was also found between TAS3a-5′D6+ and its target ARFs in *Triticum aestivum* (Tang et al., 2012). Taken together, we propose that the pathway of miR390-*TAS3*-tasiRNA- *ARF3/-4* operates in the development of longan embryo. MiR390-TAS3 tasiRNAs and ARFs define an autoregulatory network quantitatively by regulating lateral root growth (Marin et al., 2010). Here, the primary transcripts of miR390, *DlTAS3*, *DlARF3* and *-4* were all preferentially expressed in ‘Sijimi’ longan roots; therefore, we propose that their roles in roots are also conserved in longan. Besides, *DlTAS3*, *DlARF3* and *-4* were also expressed at relatively high levels in the pulp and seeds, implying extended roles in the development of longan fruit.

In summary, this study was the first to identify the longan miR390-*TAS3* tasiRNA- *ARF3/-4* axis, using a RACE strategy and the expression profiles of small RNA and their targets in longan SE. We demonstrated that *MiR390*, *DlTAS3*, *DlARF3* and *-4* define an autoregulatory network duing longan somatic embryo development.

## Author Contributions

YL participated in the study design, carried out the experimental work and wrote the manuscript. LL carried out the experimental work. ZL conceived of the study, and participated in its design and coordination and helped to draft the manuscript. LL, RL, WL, ZZ, YC, and XX prepared the materials. All authors read and approved the final version of the manuscript. All authors agree to be accountable for all aspects of the work in ensuring that questions related to the accuracy or integrity of any part of the work are appropriately investigated and resolved.

## Conflict of Interest Statement

The authors declare that the research was conducted in the absence of any commercial or financial relationships that could be construed as a potential conflict of interest.

## ABBREVIATIONS

2,4-D: 2,4-dichlorophenoxyacetic acid
ARF: auxin response factor
EC: friable-embryogenic callus
cDNA: complementary DNA
CDS: coding DNA sequence
CEs: cotyledonary embryos
GEs: globular embryos
ICpECs: incomplete compact pro-embryogenic cultures
miRNAs: microRNAs
PPRs: pentatricopeptide repeat proteins
pre-miRNA: precursor miRNA
primiRNAs: primary miRNA transcripts
RLM-RACE: RNA ligase- mediated rapid amplification of cDNA ends
RT-PCR: reverse transcription-polymerase chain reaction
RT-qPCR: real-time quantitative reverse transcription PCR
SA: salicylic acid
SE: somatic embryogenesis
Tail-PCR: thermal asymmetric interlaced PCR
tasiRNAs: trans-acting short-interfering RNAs
TEs: torpedo-shaped embryos
TSSs: transcription start sites
UTR: un-translated region
